# Seeding the Future: How Feeding Mode Shapes the Infant Gut Microbiota

**DOI:** 10.3390/microorganisms14030719

**Published:** 2026-03-23

**Authors:** Felicia Trofin, Aida Corina Badescu, Luminita Smaranda Iancu, Elena Roxana Buzila, Dana-Teodora Anton-Păduraru, Cristina Mihaela Sima, Oana-Raluca Temneanu, Anca Matei, Stefana Catalina Bilha, Ioana Alexandra Benea, Olivia Simona Dorneanu

**Affiliations:** 1 Grigore T. Popa University of Medicine and Pharmacy Iasi, 700115 Iasi, Romania; felicia.trofin@umfiasi.ro (F.T.); aida.badescu@umfiasi.ro (A.C.B.); luminita.iancu@umfiasi.ro (L.S.I.); elena-roxana.buzila@umfiasi.ro (E.R.B.); dana.anton@umfiasi.ro (D.-T.A.-P.); cristina.sima@umfiasi.ro (C.M.S.); temneanu.oana@umfiasi.ro (O.-R.T.); anca.matei@umfiasi.ro (A.M.); stefana.bilha@umfiasi.ro (S.C.B.); 2 Sf. Spiridon County Clinical Emergency Hospital Iasi, 700111 Iasi, Romania; 3 Sf. Parascheva Clinical Hospital of Infectious Diseases, 700116 Iasi, Romania; 4 National Institute of Public Health—Iasi Regional Center for Public Health, 700465 Iasi, Romania; 5 ”Sf. Maria” Children Emergency Hospital, 700309 Iasi, Romania; 6 Medical Doctoral School, “Ovidius” University, 900470 Constanta, Romania; ioana.benea@365.univ-ovidius.ro

**Keywords:** infant gut microbiota, breastfeeding, formula feeding, developmental programming window, human milk oligosaccharides (HMOs), dysbiosis, neonatal health

## Abstract

Early life represents a critical developmental programming window during which nutrition and microbial exposures shape long-term physiological function. Feeding mode is a major determinant of infant gut microbiota assembly and metabolic activity. This narrative review synthesizes current evidence comparing breastfeeding (BF) and formula feeding in relation to microbial composition, functional capacity, and immune programming during the preweaning and early postweaning periods. BF may support a relatively stable, bifidobacteria-dominated microbiota enriched in pathways involved in carbohydrate utilization, vitamin biosynthesis, and immune modulation. Human milk oligosaccharides, secretory IgA, lactoferrin, and milk-associated microbes collectively guide microbial succession, enhance barrier integrity, and support immune tolerance. In contrast, formula-fed infants typically exhibit greater microbial diversity, earlier transition toward adult-like profiles, and increased abundance of facultative anaerobes, alongside the enrichment of pathways related to bile acid and amino acid metabolism. Microbiota patterns in formula-fed infants are further influenced by formula composition, including protein load, lipid structure, and supplementation with prebiotics, probiotics, and human milk oligosaccharide analogues. Although advances in formula design have reduced compositional gaps, functional differences in microbial stability and immune programming persist. Recognizing early infancy as a sensitive programming window underscores the need for microbiome-informed nutritional strategies and longitudinal, multi-omics research to clarify causal mechanisms and optimize early-life interventions.

## 1. Setting the Context: Infant Nutrition

Early infancy represents a developmental programming window, a critical period during which biological exposures such as nutrition, microbial colonization, and environmental signals can exert lasting effects on physiological systems. During this stage, immune, metabolic, and neurodevelopmental pathways display high plasticity and are particularly sensitive to early-life inputs. Feeding mode is a key determinant of microbial assembly and metabolic signaling and may therefore influence long-term health trajectories through microbiome-mediated programming mechanisms [1].

Breastfeeding (BF) is widely regarded as the optimal feeding strategy due to its unique combination of nutrients and bioactive compounds that support immune maturation, brain development, gastrointestinal function, cardiovascular health, and cognitive outcomes [1,2,3]. Numerous studies associate BF with a lower risk of obesity, type 2 diabetes, asthma, and infections, as well as improved neurodevelopment [4,5,6,7,8,9,10,11,12,13]. Consequently, global health organizations recommend exclusive BF during the first six months of life [2,3,12].

Infant formula is designed to approximate the nutritional composition of human milk. However, it lacks many immunological and microbiota-modulating components naturally present in breast milk. Although modern formulas are supplemented with compounds such as DHA, arachidonic acid (AA), and prebiotics, they do not fully replicate the biological complexity of human milk and generally show more limited effects on immune and microbial development [14,15].

### 1.1. Motivation

Despite strong evidence supporting the health benefits of BF, the biological mechanisms underlying these effects remain incompletely understood. Human studies are often limited by confounding factors such as maternal education, socioeconomic status, maternal health, and environmental influences, all of which can affect infant outcomes [16,17]. Large cohort studies also show that both biological and psychosocial variables—beyond feeding method—are associated with behavioral and cognitive development, complicating causal interpretation [18,19,20].

Several systematic reviews have examined the relationship between feeding mode and infant gut microbiota composition. However, findings remain inconsistent due to study heterogeneity and confounding factors inherent to long-term observational research [21]. For this reason, particular attention should be given to the early postnatal period, especially the preweaning and early postweaning stages, when the effects of feeding on microbiota development are likely to be more direct and less influenced by external factors such as diet, antibiotic exposure, or environmental conditions.

**Figure 1:**
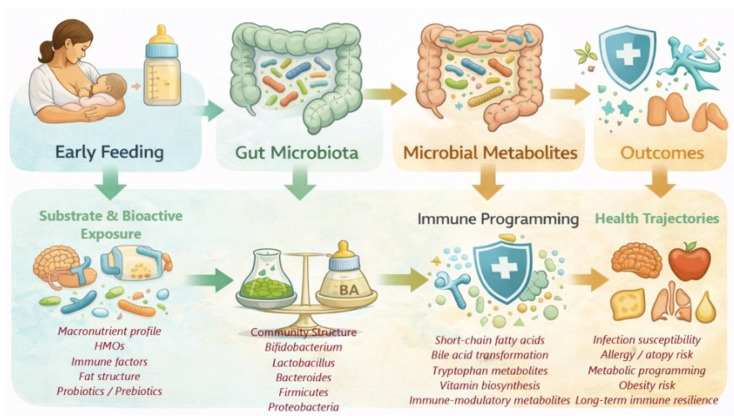
Integrative conceptual model of feeding-driven microbiome programming [16,17,18,19,20,21].

This review synthesizes current knowledge on how BF and formula feeding (FF) influence the establishment of the infant gut microbiota, highlighting the importance of early-life nutrition in shaping developmental health trajectories (Figure 1) [16,17,18,19,20,21].

### 1.2. Aim

The aim of this narrative review is to examine how BF and FF influence the development, composition, and function of the infant gut microbiota. The review focuses on the preweaning and early postweaning period (0–12 months), with particular attention to microbiota composition and functional development. It also explores potential mechanistic links between feeding mode and immune programming. By synthesizing current evidence, this review highlights how early feeding practices shape microbial colonization, immune maturation, and possible long-term health outcomes. Special attention is given to differences in microbial profiles between feeding modes and to areas where evidence remains inconsistent or limited, helping to guide future research and nutritional strategies.

### 1.3. Research Gaps

Although the health benefits of BF are well-established, the biological mechanisms through which infant feeding shapes the gut microbiota remain incompletely understood. Numerous observational studies have examined the relationship between feeding mode and microbiota composition. However, their findings are often inconsistent due to methodological heterogeneity and multiple confounding factors. Socioeconomic status, maternal education, maternal health, antibiotic exposure, and the home environment can all influence both feeding practices and infant health outcomes. These variables complicate the interpretation of microbiome data [16,17,18,19,20].

Systematic reviews investigating the relationship between BF, FF, and gut microbiota have also produced inconclusive results. This variability partly reflects differences in sampling methods, timing of microbiota assessment, and population characteristics across studies [21]. In addition, relatively few studies have focused specifically on the preweaning and early postweaning periods, when microbial colonization is most dynamic and potentially most responsive to dietary inputs.

Given the growing recognition of the gut microbiota’s role in immune development and metabolic programming, a focused synthesis of the literature during this early developmental window is needed. This review therefore examines how BF and FF differentially shape the infant gut microbiota while minimizing the influence of longer-term confounding exposures.

### 1.4. Literature Search Strategy and Study Selection

A comprehensive literature search was performed using the electronic databases PubMed and Google Scholar. The search strategy included combinations of the keywords “infant microbiota”, “BF”, “FF”, and “gut microbiome”, combined using Boolean operators such as “and” and “or” to improve search specificity.

The initial search yielded a large pool of records. After removing duplicate entries, titles were screened for relevance to the objectives of the review. Abstract screening was subsequently conducted to evaluate topical relevance, publication date, accessibility, and alignment with the scope of the review. Studies that did not address early-life microbiota development or feeding-related microbial outcomes were excluded.

Full texts of potentially eligible articles were then assessed. Additional exclusions were made due to methodological inconsistencies, limited relevance to the research question, insufficient data quality, scope misalignment, or language limitations. The selection and curation of articles for inclusion in our review adhered to rigorous criteria, ensuring alignment with our central research question: “How do BF and FF influence the infant gut microbiota during early life?”. Following this multi-step screening process, **126** studies were included in the final synthesis.

Eligible studies were qualitatively analyzed with attention to methodological rigor, clarity of results, sample size, relevance of microbiota outcomes, and overall contribution to understanding the relationship between infant feeding practices and gut microbiota development. Additional relevant articles were identified through the manual screening of reference lists from the selected studies.

## 2. Introducing the Gut Microbiota

The human gastrointestinal (GI) tract hosts a highly diverse and dynamic community of microorganisms—collectively termed the gut microbiota—comprising three domains, the Bacteria, the Archaea, and the Eucarya. This ecosystem has co-evolved with the host, forming a symbiotic relationship essential for maintaining homeostasis. The microbiome encompasses not only the microorganisms themselves, but also their genetic material, metabolic products, and the surrounding environmental conditions. In essence, the microbiome includes the microbiota plus their functional potential. Disruption of this microbial balance, known as dysbiosis, has been implicated in a wide range of intestinal and systemic diseases [22,23,24].

### 2.1. The Silent Guardian

The GI microbiota plays a vital role in maintaining host health through multiple mechanisms. It supports mucosal barrier integrity, modulates immune responses, synthesizes essential vitamins, and produces short-chain fatty acids (SCFAs) such as acetate, propionate, and butyrate, which regulate metabolism, inflammation, and epithelial homeostasis. The microbiota also enhances pathogen resistance, influences nutrient absorption, and shapes immune system maturation via microbial metabolites and host–microbe interactions. Dysbiosis of this ecosystem is linked to inflammatory, metabolic, and infectious diseases. Understanding its complex functions is key to advancing therapeutic strategies targeting microbiota-related disorders [22,23,24].

### 2.2. Architects of the Gut

While feeding mode represents a major determinant of early-life microbiota composition, it does not act in isolation. Several host and environmental factors—including mode of delivery, antibiotic exposure, maternal diet, and host genetics—interact with infant nutrition to shape microbial colonization patterns. Vaginal delivery promotes the transfer of maternal vaginal and intestinal microbes, whereas caesarean delivery is associated with delayed colonization by beneficial anaerobes such as *Bifidobacterium* and *Bacteroides*. Similarly, perinatal antibiotic exposure can disrupt early microbial succession, reducing diversity and delaying the establishment of commensal taxa. Maternal diet and metabolic status may further influence infant microbiota indirectly by altering the composition of breast milk, including fatty acids, oligosaccharides, and other bioactive components that serve as microbial substrates. In addition, emerging evidence suggests that host genetic factors may contribute to individual differences in microbial community structure and immune responses to colonizing microbes. Lifestyle and physiological factors such as physical activity, psychological stress, sleep patterns, and hormonal signaling have also been shown to influence the gut microbiota. Regular exercise can increase microbial diversity and enhance the production of beneficial metabolites, whereas chronic stress and elevated cortisol levels may disrupt microbial balance and intestinal barrier function. Similarly, sleep disturbances and circadian rhythm disruption are associated with microbial dysbiosis and altered metabolic signaling within the gut–brain axis [25,26,27,28,29,30,31,32,33]. The gut microbiota is shaped by both host and environmental factors, as seen in Table 1.

### 2.3. Mapping the Microbial Landscape of the Gut

**Figure 2:**
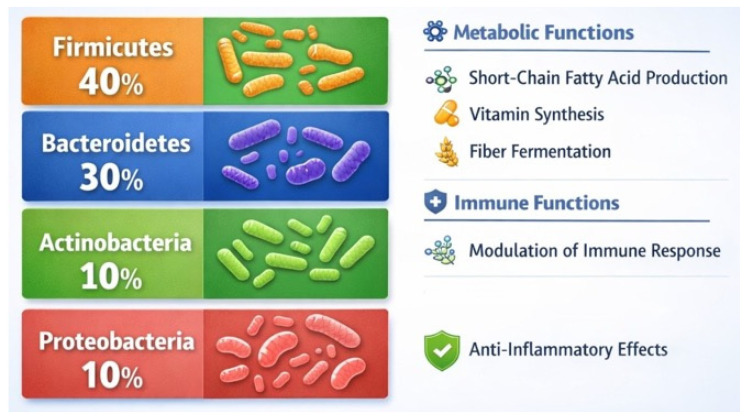
Major phyla of the gut microbiota and their function. Author’s original figure based on data from references [34,55,56,57,58,59].

Advances in sequencing technologies have significantly expanded our understanding of the gut microbiota. Landmark projects identified over 2000 human-associated microbial species, with the most representative phyla exemplified in (Figure 2). Notably, *Akkermansia muciniphila* is the sole human-representative of the *Verrucomicrobia* phylum. Despite taxonomic variation, the gut microbiota exhibits high functional redundancy, meaning that different microbial communities can perform similar physiological roles. A metagenomic analysis of over 1200 samples revealed nearly 10 million genes, with notable geographical and dietary influences shaping microbial diversity [34,55,56].

Microbial density and composition vary along the gastrointestinal tract due to chemical and immunological gradients. The small intestine, exposed to higher oxygen and antimicrobial levels, is dominated by fast-growing facultative anaerobes like *Lactobacillaceae*. In contrast, the colon hosts dense, anaerobic communities enriched in *Prevotellaceae*, *Lachnospiraceae*, and *Rikenellaceae*, which metabolize complex carbohydrates [56,57,58,59].

Although microbial taxa differ between individuals, their functional gene profiles often remain conserved. This suggests that the concept of a “functional core microbiome” may be more meaningful than a fixed taxonomic one. Efforts to classify microbiota into enterotypes—*Bacteroides*, *Prevotella*, and *Ruminococcus*-dominant profiles—highlight emerging patterns, though their validity remains debated. Understanding the structure and function of the gut microbiota is crucial for designing interventions to modulate microbial communities in health and disease (Figure 2) [22,57,59].

### 2.4. Early-Life Assembly of the Infant Gut Microbial Ecosystem

Pregnancy induces profound endocrine, immunological, and metabolic adaptations that reshape the maternal microbiota, influencing the intrauterine environment and fetal development. Gestational shifts in gut microbial composition are characterized by reduced diversity and altered abundances of key bacterial phyla in late pregnancy. Maternal microbial transmission plays a pivotal role in establishing the neonatal gut microbiome, thereby shaping early-life immune maturation, growth trajectories, and long-term health [44,60,61,62].

Mode of delivery is a key determinant of initial infant gut microbiota colonization, with vaginally delivered infants acquiring maternal vaginal and intestinal microbes, including *Lactobacillus*, at birth. Early colonization is characterized by facultative anaerobes such as *Enterobacterales* and *Staphylococcus*, which are progressively replaced by obligate anaerobes, leading to a *Bifidobacterium*-dominated (“Bifidus”) microbiota that persists until the introduction of complementary foods. Weaning drives a major ecological shift toward an adult-like microbiota enriched in *Bacteroides*, *Prevotella*, *Ruminococcus*, *Clostridium*, and *Veillonella*, accompanied by functional transitions from milk-derived substrate utilization to complex carbohydrate metabolism, vitamin synthesis, and xenobiotic degradation. By approximately three years of age, the gut microbiota attains an adult-like configuration, shaped by early-life exposures including delivery mode, BF, antibiotic use, and environmental factors (Table 2) [42,44,45,63,64,65,66,67].

### 2.5. Microbiome–Host Interactions in Infancy

**Figure 3:**
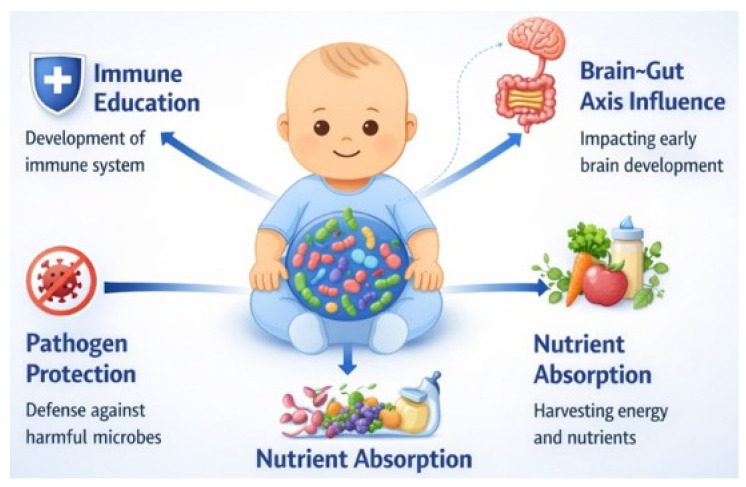
Microbiota’s impact on infant health. Author’s original figure based on data from reference [42].

The gut microbiota plays a fundamental role in human health from birth, contributing to immune system maturation, central nervous system (CNS) development, and nutrient digestion and metabolism. Early life represents a critical window for microbial colonization, during which perturbations can have lasting consequences for health across the lifespan. During the first two years of life, rapid brain growth coincides with the establishment of the gut microbiota, highlighting a close temporal and functional relationship between microbial colonization and neurodevelopment. Gut microbiota influence brain development through multiple pathways, including the production of bioactive metabolites such as SCFAs, modulation of neurotransmitter synthesis, regulation of microglial maturation, and maintenance of blood–brain barrier integrity. These microbiota-derived signals contribute not only to structural and functional brain development but also to early-life behavior and cognitive outcomes (Figure 3) [68].

**Figure 4:**
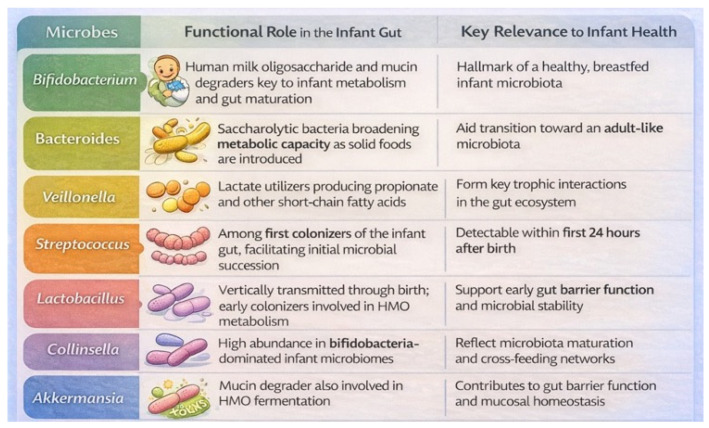
Functional role of bacteria in the infant gut. Author’s original figure based on data from reference [42].

In parallel, the infant gut microbiota plays a central role in shaping the immune system. Following birth, microbial colonization of mucosal surfaces occurs alongside immune maturation, promoting immune tolerance while enabling effective defense against pathogens. Microbial metabolites interact with host immune cells through specific receptors, including G protein-coupled receptors, aryl hydrocarbon receptor, and Toll-like receptors, thereby regulating both innate and adaptive immune responses. Disruptions to microbial colonization during this sensitive period have been associated with increased susceptibility to immune-mediated and metabolic diseases later in life, including asthma, allergies, inflammatory bowel disease, and diabetes. Collectively, current evidence underscores early-life gut microbiota as a key determinant of long-term neurological, immunological, and metabolic health, emphasizing the importance of understanding microbial colonization patterns and mechanisms to inform preventive and therapeutic strategies (Figure 4) [68].

## 3. Breastfeeding: Nature’s First Functional Food

Human breast milk is a complex and dynamic biological fluid that provides essential macronutrients and bioactive components critical for infant growth and development. Its composition varies with lactation stage and feeding sessions to meet the infant’s changing needs, maternal diet, genetics, environment, and milk handling, reflecting both biological adaptability and complexity [69,70,71,72].

Beyond water, its primary components include carbohydrates, proteins, and fats. Lactose is the predominant carbohydrate, supporting energy demands, osmotic balance, mineral absorption, and brain development, while protein fractions—particularly whey proteins—deliver essential amino acids and immune-related bioactivity. Milk lipids, accounting for nearly half of total caloric intake, are critical for central nervous system maturation and retinal development, largely through long-chain polyunsaturated fatty acids such as DHA [70,71,72,73,74,75]. In addition to macronutrients, it contains a wide range of immunologically active components, including secretory IgA, lactoferrin, lysozyme, cytokines, and human milk oligosaccharides (HMOs), which collectively promote immune maturation and protect against infection. Together, the nutritional and immunological components of HBM provide both immediate and long-term health benefits, underscoring BF as a cornerstone of infant nutrition and disease prevention [75,76,77,78].

Extensive evidence demonstrates that due to its composition, breast milk significantly reduces infant morbidity and mortality, lowering the risk of infectious, metabolic, immune-mediated, and chronic diseases [74,75,76,77,78].

### 3.1. Human Milk Microbiome: Seeding the Infant Gut

Human breast milk was originally assumed to be free of microorganisms [79], and the detection of bacteria was traditionally interpreted as a sign of contamination or infection, particularly in cases of mastitis or mother-to-infant pathogen transfer [52]. This assumption was overturned in the early 2000s, when both culture-based and molecular approaches consistently demonstrated the presence of commensal microbes in breast milk [80,81]. Initial studies identified mainly Gram-positive bacteria, while later work revealed the presence of lactic acid-producing species, indicating that BF may actively supply beneficial microorganisms to the infant gastrointestinal tract [80]. The application of next-generation sequencing technologies has since confirmed that breast milk contains complex and diverse microbial communities that contribute to early gut colonization [82].

Although the microbial profile of breast milk varies considerably, many studies report a relatively stable core microbiota dominated by genera such as *Staphylococcus*, *Streptococcus*, *Bifidobacterium*, *Lactobacillus*, and *Corynebacterium* [82]. Variability in reported core taxa likely arises from differences in maternal characteristics, geographic and environmental factors, lactation stage, antibiotic exposure, as well as sampling and analytical methods [81]. Nevertheless, converging evidence indicates that the breast milk microbiome plays a functional role in establishing and shaping the infant gut microbial ecosystem. The detection of shared bacterial strains in maternal milk and infant feces further supports direct microbial transmission during BF [83,84,85].

**Figure 5:**
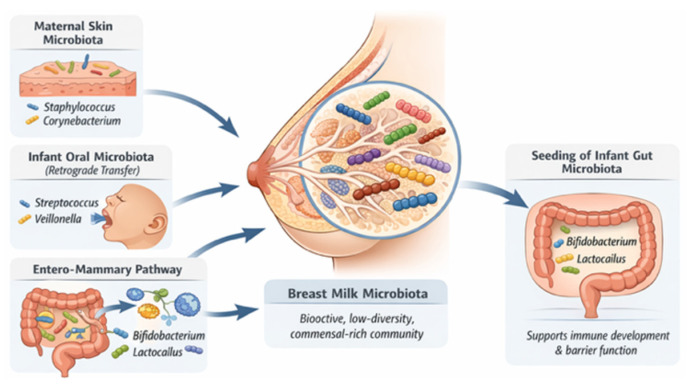
Breast milk-associated microbes. Created by the authors based on references [53,54,55,56,57,58,59,60,61].

Exclusively BF infants commonly exhibit a bifidobacteria-enriched gut microbiota. Beyond viable bacteria, breast milk also contains bacteria-derived extracellular vesicles that may influence host–microbe communication and immune signaling pathways. The origins of breast milk–associated microbes appear to be multifactorial, involving contributions from maternal skin, the infant oral cavity, and microbial translocation from the maternal gut through the enteromammary pathway (Figure 5) [80,81,82,83,84,85,86,87].

Importantly, the relevance of the breast milk microbiome extends beyond infant health, as microbial imbalances have been associated with maternal conditions such as mastitis and may also be linked to breast cancer risk. Together, these findings position the breast milk microbiome as a critical biological interface connecting maternal and infant health, with implications for microbial colonization, immune programming, and disease susceptibility. Further progress in this field will depend on standardized methodologies and mechanistic studies to clarify causal relationships and therapeutic potential [87].

### 3.2. Hidden Drivers of Infant Microbiota

**Figure 6:**
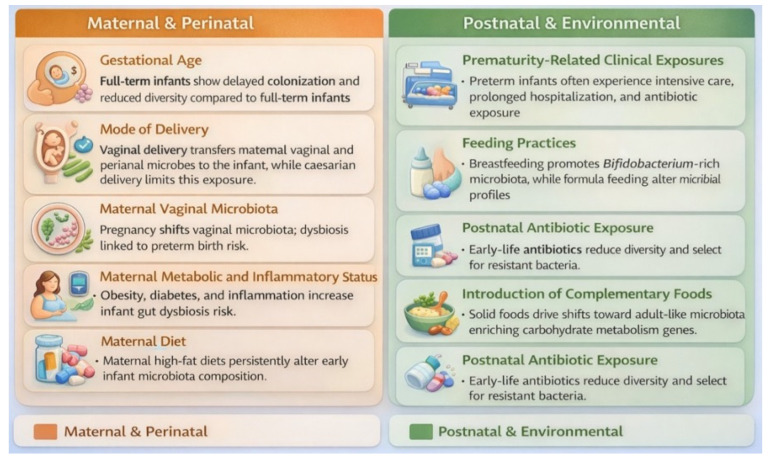
Factors that influence the infant microbiota [63,64,65,66,67,68,69,70,71,72].

Vaginal delivery and BF promote early colonization by beneficial bacteria such as *Bifidobacterium* and *Lactobacillus*. This is supported by maternal microbial transfer and bioactive components in human milk. In contrast, caesarean section, formula feeding, and antibiotic exposure delay microbial maturation. They also reduce anaerobic diversity and increase potentially pathogenic bacteria (Figure 6). Preterm birth further disrupts normal colonization patterns through clinical exposures and delayed establishment of beneficial microbes, increasing susceptibility to immune dysregulation and adverse outcomes (Figure 6). As solid foods are introduced, the microbiota undergoes functional and compositional shifts toward an adult-like configuration, with cumulative early-life exposures exerting lasting effects on immune development and long-term health trajectories [35,36,46,48,68,88,89,90,91,92]. BF exclusivity and duration influence the infant gut microbiome, with dose-dependent effects on microbial composition (Figure 6) [93].

### 3.3. Milk as a Modulator of Infant Microbiota

Human milk is a complex and dynamic fluid composed primarily of water, with key macronutrients including lactose, fat, and proteins, which together supply most of the infant’s energy. Lactose remains relatively constant during lactation, contributing to energy provision, osmotic balance, and mineral absorption, while proteins are present as easily digestible whey and casein fractions enriched with bioactive components such as lactoferrin, α-lactalbumin, and secretory immunoglobulin A (sIgA). Human milk also provides essential vitamins and minerals sufficient for normal infant growth, with the exception of vitamins D and K [94].

HMOs are structurally diverse, reach the infant colon intact and selectively shape the gut microbiota. HMOs function as prebiotics that support beneficial gut microbiota, enhance intestinal barrier function, and reduce pathogen adhesion, particularly in early life. They are preferentially utilized by specific *Bifidobacterium* species, which possess specialized gene clusters enabling efficient HMO metabolism and competitive dominance in the infant gut. Fermentation of HMOs by bifidobacteria produces organic acids and short-chain fatty acids that lower gut pH, inhibit pathogen growth, support epithelial barrier integrity, and may support immune maturation. Bifidobacteria-derived metabolites further contribute to the anti-inflammatory signaling and regulation of both innate and adaptive immune responses in early life. Together, these mechanisms position HMOs and HMO-adapted bifidobacteria as central drivers of intestinal immune development and protection against inflammation and infection in infancy [49,88,95,96].

Human colostrum and mature milk are rich in sIgA, which is actively transported across the mammary epithelium and dominates antibody-mediated protection at mucosal surfaces. sIgA is highly resistant to intestinal degradation and functions primarily through immune exclusion, agglutinating microbes and toxins, inhibiting epithelial adhesion, and limiting inflammatory signaling in the neonatal gut. Extensive observational studies link higher milk sIgA levels to reduced risk of enteric and respiratory infections, as well as protection against diseases such as diarrhea, necrotizing enterocolitis, and allergic disorders. Beyond pathogen defense, sIgA plays a central role in shaping the infant gut microbiota by selectively coating commensal bacteria, promoting beneficial colonization patterns, and fostering immune tolerance. Together, milk-derived sIgA acts as a critical maternal immunological bridge that supports early microbial homeostasis and immune development during a period of heightened infant vulnerability [49,50,88,95,96,97,98,99].

### 3.4. Microbiota Composition in BF Infants

**Figure 7:**
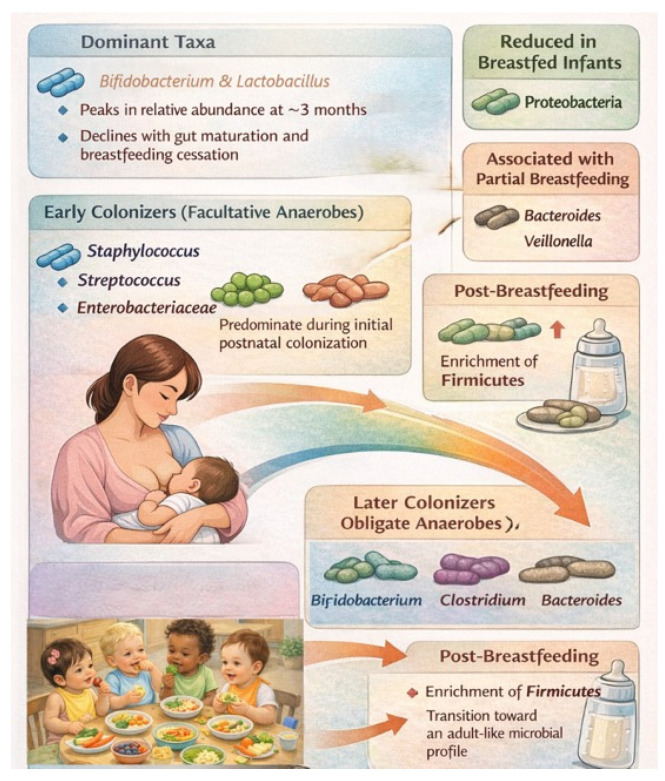
Evolution of the microbiota during and after breastfeeding [74,75,76,77,78,79].

High-throughput sequencing studies show that BF infants harbor distinct microbial profiles enriched in commensal taxa such as *Bifidobacterium* and *Lactobacillus*, while human milk itself contains a diverse microbiota that contributes directly to early gut colonization. Emerging evidence supports a gut–lactation pathway in which maternal microbes and milk-derived metabolites shape neonatal gut microbiota and intestinal health, although key questions remain regarding colonization dynamics and long-term outcomes (Figure 7) [95,96,97,98,99].

Early microbial succession typically begins with facultative anaerobes, including *Staphylococcus*, *Streptococcus*, and *Enterobacteriaceae*, followed by obligate anaerobes such as *Bifidobacterium*, *Clostridium*, and *Bacteroides*. BF infants also tend to harbor higher abundances of *Lactobacillus* and lower levels of *Proteobacteria* and adult-associated taxa, although findings vary across studies. Both BF exclusivity and duration exert dose-dependent effects on gut microbiota composition, with partial BF associated with earlier expansion of genera such as *Bacteroides* and *Veillonella*. Cessation of BF, more than the introduction of solid foods, may influence a shift toward a more diverse, *Firmicutes*-enriched microbiota. Beyond taxonomic composition, BF influences microbial functional capacity, with an enrichment of genes involved in carbohydrate and lipid metabolism, vitamin biosynthesis, and energy production (Figure 7) [97,98,99].

Metabolomic studies further distinguish BF and FF infants, with BF associated with altered bile acid profiles and higher levels of anti-inflammatory metabolites. Associations between BF and fecal SCFAs are variable, likely reflecting rapid absorption and microbial cross-feeding rather than true differences in production. Collectively, these findings demonstrate that BF shapes not only the structure but also the functional trajectory of the infant gut microbiome during a critical developmental window (Figure 7) [49,88,95,96]. Early feeding may influence metabolic programming through effects on microbial colonization, metabolite production, and host signaling pathways, but the long-term causal contribution of these mechanisms requires further study.

## 4. Infant Formula: Engineered Nutrition for Early Life

Commercial infant formulas are used worldwide as partial or complete substitutes for breast milk in infants and young children up to 36 months. These products are typically categorized into infant formula (0–6 months), follow-up formula (6–12 months), and toddler formula (13–36 months), and are manufactured from milk proteins, carbohydrates, vegetable oils, micronutrients, and added bioactive components. Infant formulas are primarily intended for situations in which BF is not possible, while follow-up and toddler formulas are considered nutritionally nonessential. Available in powdered, liquid concentrate, and ready-to-feed forms, formulas vary substantially in cost and preparation requirements. The global infant formula market continues to expand, driven by demographic, socioeconomic, and lifestyle changes, highlighting the growing reliance on FF in early life nutrition [37,94,100,101,102].

### 4.1. Formula Feeding Nutrition Design

Infant formulas are designed to deliver balanced macronutrients and micronutrients, typically combining milk-derived proteins (e.g., whey/casein fractions or hydrolysates) [103], carbohydrates (often lactose, sometimes maltodextrin or glucose polymers) [104], vegetable oil-based fats [105], and vitamin–mineral fortification [94,101,103]. From a microbiome perspective, the most influential ingredients are the carbohydrate fraction [104] and any added prebiotics/probiotics [94]. Unlike most digestible sugars, non-digestible oligosaccharides added to some formulas (e.g., GOS/FOS and select HMOs such as 2′-FL and LNnT) can reach the colon and selectively promote bifidobacteria while limiting pathogen adhesion [94,104]. The protein source and degree of hydrolysis may also shift gut ecology by changing peptide availability and gastric/intestinal digestion kinetics [103]. Fat composition (including the presence of DHA/ARA and structured triglycerides) can indirectly influence microbiota through effects on bile acid flow and intestinal inflammation. Minerals and vitamins support infant growth but may also affect gut conditions (e.g., osmolarity, redox balance) that shape microbial niches [87]. Many products now include bioactive add-ons (e.g., lactoferrin, MFGM components, nucleotides) intended to narrow functional gaps with human milk and potentially modulate microbial colonization. Formula composition also varies substantially across brands and regions, so microbiota outcomes can differ even among FF infants. Overall, formula-driven microbiome effects reflect how specific nutrients and bioactives alter substrate availability and gut physiology during early colonization [37,94,100,101,102,103,104,105].

### 4.2. Nutritional Shaping of the Infant Gut Microbiome

Feeding type strongly shapes microbial metabolic functions, with exclusive BF linked to pathways involved in vitamin synthesis, lipid metabolism, and detoxification, whereas FF feeding favors carbohydrate metabolism and higher SCFA production. Macronutrient composition of infant FF substantially influences microbial composition. Whey-predominant FF, lipid structure, and triglyceride configuration can shift the microbiota toward profiles resembling BF infants, particularly by increasing *Bifidobacterium* and *Lactobacillus* [37,94,100,101,102,103,104,105,106].

Probiotic and symbiotic supplementation can accelerate microbiome maturation and promote anti-inflammatory metabolic outputs, though effects vary by strain and formulation. Conversely, certain additives, emulsifiers, excess minerals, and processing-induced changes may disrupt microbial balance and contribute to dysbiosis. Overall, early nutrition is a dominant determinant of gut microbiome structure and function, with lasting implications for immune and metabolic health. Continued research integrating microbial composition, function, and processing effects is essential to optimize formula design that more closely supports the microbiome trajectory seen in BF infants [37,38,94,96,100,101,102,103,104,105,106,107,108].

### 4.3. Microbial Profiles of FF Infants

FF infants harbor a gut microbiota that differs markedly from that of BF infants, characterized by greater diversity, reduced stability, and earlier maturation toward an adult-like configuration. These microbial communities show reduced dominance of *Bifidobacterium* and depletion of other infant-associated commensals such as *Bacteroides* and *Parabacteroides*, alongside expansion of facultative anaerobes including *Enterobacteriaceae*, *Staphylococcaceae*, and *Enterococcaceae*. FF is also associated with the enrichment of metabolic pathways related to bile acid, amino acid, and nucleotide metabolism. Several potentially pathogenic species, such as *Clostridioides difficile*, *Klebsiella pneumoniae*, and *Staphylococcus aureus*, are more prevalent in FF infants. Notably, these microbial shifts are accompanied by higher abundances of antibiotic resistance genes, largely driven by the expansion of *Enterobacteriaceae*, which may increase vulnerability to adverse outcomes such as necrotizing enterocolitis [106].

## 5. Advancements and Clinical Implications of Infant Formula

Although BF remains the recommended standard for infant nutrition, infant formula plays an essential role when BF is not possible or insufficient. Recent technological advancements in formula design aim to better replicate the functional properties of human milk. Modern formulations increasingly incorporate bioactive components such as HMOs, probiotics, prebiotics, lactoferrin, and milk fat globule membrane fractions, which have been shown to support beneficial microbial colonization and immune development. Clinical studies indicate that formulas supplemented with HMOs, such as 2′-fucosyllactose and lacto-N-neotetraose, can promote the growth of *Bifidobacterium* species and partially shift the gut microbiota toward profiles observed in BF infants. In neonatal intensive care settings and in cases where BF is contraindicated or limited, FF remains a critical nutritional intervention that supports adequate growth and development. Continued improvements in formula composition, informed by advances in microbiome science and human milk research, may further narrow the functional gap between formula and human milk while providing safe and effective nutrition for infants who cannot be BF [53,54,109,110,111,112].

### 5.1. Distinct Gut Microbiota in Exclusive vs. Non-Exclusive BF

There are consistent differences in gut microbiota composition between exclusively BF and non-exclusively BF infants during the first six months of life. Compared with exclusively BF infants, non-exclusively BF infants exhibit higher microbial diversity, increased microbiota maturity, and bacterial communities that resemble an adult-like profile at an earlier age. In contrast, exclusively BF is associated with a more stable and less diverse gut microbiota, which may be better aligned with the immunological and physiological immaturity of early infancy [97]. Across populations, non-exclusively BF infants show increased relative abundances of *Firmicutes* and *Bacteroidetes*, including genera such as *Bacteroides*, *Eubacterium*, and *Veillonella*, whereas exclusively BF infants are consistently enriched in *Bifidobacterium* [97,98]. These compositional differences are accompanied by functional shifts, with non-exclusively BF microbiota showing increased pathways related to carbohydrate metabolism and reduced pathways involved in lipid metabolism, vitamin biosynthesis, and detoxification. Such functional profiles may help explain the higher risk of obesity, metabolic disorders, and immune-mediated diseases associated with reduced BF duration [99].

Notably, the effects of non-exclusively BF on gut microbiota are modified by mode of delivery, with caesarean-delivered infants showing greater microbial perturbations than vaginally delivered infants. In these infants, FF appears to exacerbate microbiota immaturity and depletion of key bacterial groups, highlighting the particular importance of exclusively BF following caesarean birth [49,50,95]. Differences between exclusively BF and non-exclusively BF microbiota persist beyond six months of age, with shorter exclusively BF duration associated with earlier loss of *Bifidobacteriaceae* dominance. Moreover, exclusively BF exerts a protective effect during diarrheal episodes, preserving microbial diversity and community structure and limiting pathogen-associated shifts. Collectively, these findings indicate that exclusively BF supports a homeostatic developmental trajectory of the infant gut microbiota, with implications for both short- and long-term health outcomes [49,50,88,95,96].

### 5.2. Distinct Infant Gut Microbiota in BF vs. FF Infants

**Figure 8:**
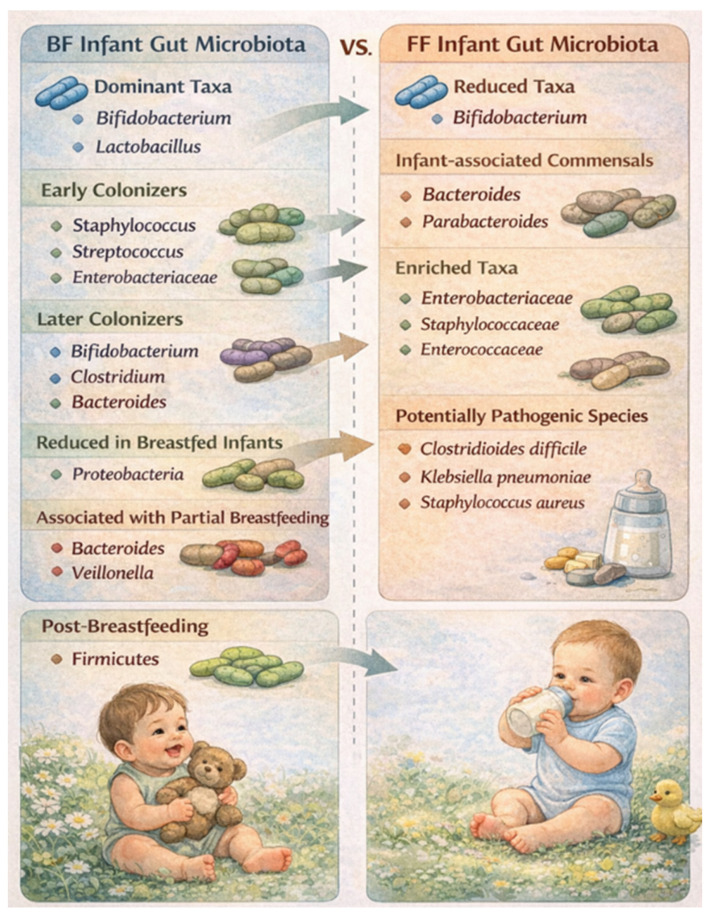
BF infant gut microbiota vs. FF infant gut microbiota.

BF infants exhibited lower gut microbial alpha diversity than FF infants during early life, reflecting dominance of infant-adapted taxa rather than microbial immaturity. Across both feeding methods and time points, *Bifidobacterium* was the predominant genus, with *Enterobacteriaceae* as a common secondary colonizer. At 40 days of age, BF infants showed significantly higher relative abundances of *Bifidobacterium* and *Bacteroides*, alongside lower levels of *Streptococcus*, *Enterococcus*, *Veillonella*, *Clostridioides*, and *Lachnospiraceae* compared with FF infants. Over time and following the introduction of solid foods, *Bifidobacterium* and *Enterobacteriaceae* declined across feeding groups, consistent with microbiota maturation. FF infants displayed greater microbial diversity and increased representation of adult-associated taxa, including *Clostridia* and *Veillonella* (Figure 8) [113].

## 6. Discussion

The differential microbial patterns observed between BF and FF occur within a defined developmental programming window, during which immune tolerance, metabolic regulation, and epithelial barrier function are highly plastic. Microbiota-mediated signaling during this period may exert disproportionate long-term effects compared to similar exposures later in life. Thus, early nutritional modulation of the gut ecosystem may represent a critical lever for influencing lifelong disease susceptibility.

Comparative studies examining gut microbiota development in exclusively BF versus FF infants remain limited and are often constrained by small cohort sizes. Available evidence nevertheless indicates that infant gut microbial composition differs substantially by feeding mode and varies further according to formula type, highlighting the importance of formula composition in shaping early microbial trajectories.

BF is thought to support the composition and functional maturation of the infant gut microbiota, with important implications for immune development and disease susceptibility. Infants who are exclusively BF typically harbor a microbiota characterized by low overall diversity but high stability, dominated by infant-adapted taxa such as *Bifidobacterium*. This microbial configuration reflects selective pressures imposed by human milk components, particularly HMOs, and appears well-suited to the immunological immaturity of early life. In contrast, partial or absent BF is associated with accelerated microbiota maturation, increased diversity, and early expansion of adult-associated taxa, which may represent a deviation from optimal developmental trajectories.

The functional consequences of BF-associated microbial profiles extend beyond taxonomic composition. BF may support the enrichment of microbial pathways involved in SCFA production and immune modulation while limiting the expansion of potentially pro-inflammatory *Proteobacteria*. These metabolic features support intestinal barrier integrity, immune tolerance, and controlled inflammatory responses. Importantly, the predominance of bifidobacteria in BF infants has been consistently associated with reduced risk of enteric infection, allergic disease, and immune dysregulation in later life [39].

Beyond feeding composition, early-life dietary practices strongly influence microbiome maturation. Exclusive milk feeding during the first six months supports microbiome stability, while the gradual introduction of complementary foods facilitates a controlled transition toward a more adult-like microbial community. Overall, early nutrition remains a dominant determinant of gut microbiome structure and function, with lasting implications for immune and metabolic health. Importantly, microbial diversity in early infancy does not necessarily indicate a healthier microbiome, as infant-adapted ecosystems are naturally low-diversity and functionally specialized.

Infant formula remains clinically indicated in situations where BF is contraindicated or not feasible, including maternal illness, insufficient milk supply, certain metabolic disorders, preterm birth requiring specialized nutrition, or maternal use of medications incompatible with BF. For infants who cannot be BF, emerging evidence suggests that microbiome-supportive formula design may partially bridge the gap. Lactose-based formulas appear preferable, as lactose serves both as an energy source and a prebiotic substrate that supports bifidobacterial growth. Protein quantity and quality are also critical, as excessive protein intake early in life may promote unfavorable microbial fermentation and metabolic dysregulation, whereas protein levels closer to human milk better support balanced microbial development. Lipid structure further influences microbiota composition, with formulas enriched in sn-2 palmitate associated with increased bifidobacteria, softer stools, and improved gastrointestinal tolerance. Prebiotic fibers may further promote beneficial taxa when provided at physiologically relevant concentrations [53,54,109,110,111,112].

Technological advancements in formula design—such as the incorporation of HMOs, optimized protein profiles, structured lipids, probiotics, and bioactive components—reflect efforts to more closely approximate the functional properties of human milk, particularly in supporting gut microbiota development and immune maturation [51]. From an industry perspective, ongoing innovation is driven by emerging microbiome research, regulatory standards, and growing consumer demand for evidence-based, microbiome-supportive formulations, positioning precision nutrition as a central focus of future product development [53,54,109,110,111,112].

A lower-diversity microbiota in early infancy should not be interpreted using the same ecological framework applied to adults. In the infant gut, relatively low diversity often reflects a developmentally appropriate, highly specialized microbial ecosystem rather than impaired health. Early life is characterized by a limited and selective nutrient environment, particularly in BF infants, in whom human milk oligosaccharides provide a dominant substrate that strongly favors HMO-adapted taxa such as *Bifidobacterium*. This ecological filtering promotes a microbiota that is less diverse but metabolically efficient and functionally aligned with the needs of the immature host. Such specialization supports key early-life processes, including epithelial barrier maturation, colonization resistance, immune tolerance, and production of metabolites involved in host–microbe signaling. In contrast, a more diverse microbiota in FF infants may reflect the earlier expansion of adult-associated or facultative taxa, but this does not necessarily indicate a more beneficial or mature state for the infant host. Thus, in infancy, microbial specialization and stability may be more informative indicators of gut ecosystem suitability than diversity alone [40,41,88,114,115].

Collectively, the findings support the concept that BF establishes a homeostatic, infant-optimized gut ecosystem that supports healthy immune programming. While variability in maternal, environmental, and methodological factors complicates direct comparisons across studies, the consistency of observed patterns across diverse populations underscores the biological relevance of BF-influenced microbiota development. To avoid overstatement, many of the proposed links between early feeding, microbiota development, and later health outcomes should be interpreted as biologically plausible associations rather than definitive causal relationships. Current evidence supports the view that BF is associated with a microbiota enriched in infant-adapted taxa and metabolites relevant to epithelial and immune function; however, the extent to which these microbial patterns directly mediate long-term disease protection, metabolic programming, or immune tolerance remains incompletely resolved. Much of the available evidence derives from observational cohorts, which are inherently vulnerable to confounding by maternal, environmental, and clinical factors. Accordingly, while early feeding clearly shapes microbiota assembly during a sensitive developmental window, stronger mechanistic and longitudinal studies are needed to define the durability and clinical significance of these effects.

Differences observed between BF and FF infant microbiota should be interpreted within a broader ecological context, as feeding practices interact with multiple biological and environmental factors that shape early microbial colonization. A strong consensus exists that early-life nutrition influences gut microbiota development, with BF infants typically exhibiting communities enriched in *Bifidobacterium* and other taxa specialized in human milk oligosaccharide metabolism. Feeding practices also appear to affect microbial metabolic functions, including carbohydrate fermentation, immune modulation, and short-chain fatty acid production. However, several aspects remain debated. Reported differences in microbial diversity between BF and FF infants vary across studies, and the long-term health implications of early feeding-associated microbial patterns remain incompletely understood. Part of this variability likely reflects methodological differences rather than true biological inconsistency. Earlier studies frequently relied on culture-based techniques or 16S rRNA sequencing, which provide limited taxonomic resolution, whereas newer shotgun metagenomics approaches offer improved species-level and functional insights. Additional variability arises from differences in sampling time points, definitions of BF exclusivity, geographic and lifestyle factors, maternal diet, and heterogeneity in formula composition. Analytical pipelines and bioinformatics processing methods can also influence the reported microbial profiles. Recognizing these methodological and ecological influences is essential for interpreting current evidence and highlights the need for standardized protocols and longitudinal multi-omics approaches to better define feeding-related microbiome trajectories (Table 3) [40,51,96,114,116,117].

Early-life gut dysbiosis has been consistently associated with the development of atopic dermatitis, food allergy, asthma, and multisensitized atopy, likely through impaired regulatory T cell induction and barrier dysfunction. Beneficial taxa such as *Bifidobacterium* and members of the *Clostridia* class are linked to immune tolerance, in part through the production of SCFAs that support epithelial integrity and anti-inflammatory immune pathways. Reduced SCFA levels and altered microbial metabolic capacity in infancy have been associated with increased allergic disease risk across multiple cohorts. Although epidemiological evidence suggests that exclusive BF may protect against allergic outcomes by fostering a microbiome enriched in infant-adapted bacteria, methodological limitations and ethical constraints continue to complicate causal inference [113,118].

The findings of this review have several important clinical implications. First, in parental counselling, healthcare providers should communicate that early feeding choices influence microbial development during a critical window of immune programming, while also acknowledging that optimized formula options can support microbiome maturation when BF is not feasible. Second, in the Neonatal Intensive Care Unit setting—particularly for preterm or caesarean-delivered infants—strategies that promote early colonization with beneficial taxa (e.g., human milk provision, judicious probiotic use where evidence supports it) may reduce dysbiosis-associated complications such as necrotizing enterocolitis. Third, antibiotic stewardship in early life is essential, as unnecessary exposure may disrupt microbiota assembly and increase long-term immune and metabolic risk. Finally, continued refinement of infant formula design—including physiologically relevant oligosaccharides, appropriate protein quantity and quality, and optimized lipid structures—represents a translational opportunity to better support microbiome-mediated immune development. Collectively, integrating microbiome science into clinical decision-making may improve both short- and long-term health outcomes in infants across diverse feeding contexts.

To avoid overstatement, many of the proposed links between early feeding, microbiota development, and later health outcomes should be interpreted as biologically plausible associations rather than definitive causal relationships. Current evidence supports the view that BF is associated with a microbiota enriched in infant-adapted taxa and metabolites relevant to epithelial and immune function; however, the extent to which these microbial patterns directly mediate long-term disease protection, metabolic programming, or immune tolerance remains incompletely resolved. Much of the available evidence derives from observational cohorts, which are inherently vulnerable to confounding by maternal, environmental, and clinical factors. Accordingly, while early feeding clearly shapes microbiota assembly during a sensitive developmental window, stronger mechanistic and longitudinal studies are needed to define the durability and clinical significance of these effects.

### Future Directions

Recent advances in longitudinal cohort studies and multi-omics approaches have substantially refined our understanding of infant gut microbiome development, revealing dynamic interactions between microbial composition, metabolic function, and host immune signaling during early life. These integrative analyses provide higher-resolution insights into microbiome–host crosstalk and highlight the potential of early nutritional interventions to modulate developmental trajectories with implications for long-term health outcomes [119,120,121,122,123,124,125,126].

Future research should move beyond descriptive comparisons of BF and formula feeding toward mechanistic and translational studies that clarify causal pathways linking early nutrition, microbial development, and health outcomes. Longitudinal, multi-omics approaches integrating metagenomics, metabolomics, immune profiling, and host gene expression will be critical to define functional consequences of feeding-associated microbial shifts. Greater emphasis should be placed on identifying specific human milk components and formula additives that drive beneficial microbial and immunological responses, as well as on defining sensitive developmental windows when nutritional interventions are most effective. Carefully designed interventional studies, including ethically appropriate supplementation trials and natural experiments, may help disentangle feeding effects from confounding factors. Future research should focus on elucidating causal mechanisms, identifying critical milk–microbe interactions, and determining how BF duration and exclusivity can be optimized to support long-term health. Finally, harmonized protocols, diverse global cohorts, and long-term follow-up will be essential to translate microbiome insights into precision nutrition strategies that support optimal infant health across populations.

## 7. Conclusions

Early-life nutrition acts within a critical developmental programming window to shape the infant gut microbiota, immune maturation, and long-term metabolic health. Recognizing infancy as a biologically sensitive programming period highlights the importance of optimizing early feeding strategies to support durable health trajectories. FF is often associated with a more diverse and rapidly maturing microbiome, though the magnitude and direction of these effects vary by formula composition and study design, often enriched in facultative anaerobes and adult-like metabolic functions. Recent advances in formula composition, including the addition of human milk oligosaccharides, probiotics, and optimized protein and lipid structures, have narrowed—but not eliminated—the gap with human milk. Continued research integrating microbiome composition, function, and long-term health outcomes is essential to further optimize infant nutrition. These insights provide a framework for developing microbiome-informed feeding strategies that better support infant health when BF is not possible.

## Figures and Tables

**Table 1:** Factors influencing gut microbiota development and composition.

Factor	Description	Impact on Gut Microbiota	Ref.
Diet	Intake of fiber, fats, proteins, and carbohydrates, and the diversity of food types consumed.	Directly affects microbial diversity, abundance, and metabolism; high-fiber diets promote beneficial bacteria, while high-fat/sugar diets may increase pathogenic microbes.	[22,23,24,34,35,36,37,38,39]
Age	Developmental changes from birth to old age, including weaning, childhood, adolescence, and aging.	Newborns have a less diverse microbiota that evolves over time; its shifts occur with aging, often reducing diversity and increasing opportunistic pathogens.	[22,23,24,34,40,41]
Genetics	Host genetics influencing immune responses, intestinal permeability, and predisposition to diseases.	Certain genetic predispositions can shape microbial populations, with genetic factors affecting immune tolerance to gut microbes and altering gut-barrier function.	[22,23,24,34,41,42,43]
Type of Birth (Vaginal vs. C-Section)	The method of delivery influences initial microbial exposure.	Vaginal births expose infants to maternal microbes, fostering a diverse microbiota; C-sections lead to a less diverse initial microbiota, potentially affecting immune development.	[22,23,24,34,44,45,46,47]
Antibiotic Use	The use of antibiotics alters microbial balance by eliminating sensitive species.	Short-term antibiotic use can reduce microbial diversity; prolonged or frequent use can lead to long-lasting shifts, potentially promoting antibiotic-resistant bacteria.	[22,23,24,34,36,48,49,50,51]
Infections & Diseases	Infections (e.g., GI or systemic) or chronic diseases (e.g., diabetes, etc.) impact microbiota composition.	Pathogenic infections can disrupt microbiota, leading to dysbiosis (imbalanced microbial community), which may contribute to disease exacerbation.	[22,23,24,34,52]
Environment	Geographical location, living conditions, exposure to animals, hygiene practices, and urban vs. rural living.	Environmental exposure affects microbial diversity, with rural environments often hosting a greater variety of microbes compared to urban settings.	[22,23,24,25,26,34,53]
BF vs. FF	Feeding type during infancy (breast milk vs. formula milk).	Breast milk may support a more diverse microbiota due to its prebiotics and immune factors, while FF can lead to a less diverse microbiome in early life.	[22,23,24,26,34,39]
Probiotics and Prebiotics	The intake of probiotic foods/supplements (live beneficial bacteria) and prebiotics (dietary fibers feeding gut microbes).	Probiotics can enrich beneficial microbial populations, while prebiotics support gut health by stimulating beneficial microbes and enhancing gut function.	[22,23,24,34,53,54]
Medication & Hormones	Use of medications like antacids, proton pump inhibitors, or hormone therapies.	Certain medications or hormonal changes can alter gut permeability and microbial populations, potentially leading to dysbiosis or affecting metabolic processes.	[13,22,23,24,30,31,32,33,34]
Social Interactions	Sharing microbes with others, particularly in family or community settings, can impact microbiota composition.	Close physical interactions and shared environments can lead to microbiome similarities, influencing microbial diversity and stability.	[22,23,24,25,26,34,53]

Abbreviations: GI = gastrointestinal, Ref. = references BF = breastfeeding, FF = formula-feeding.

**Table 2:** Key microbial taxa associated with different infant life stages.

Age Bracket	Dominant Microbial Taxa	Key Characteristics & Notes	Associated Factors
Birth (0 days)	*Staphylococcus*, *Enterobacteriaceae*, *Lactobacillus*, *Streptococcus*,	Facultative anaerobes dominate due to oxygen-rich environment; low diversity	Mode of delivery (vaginal vs. C-section), maternal microbiota
1 Month	*Bifidobacterium*, *Lactobacillus*, *Enterococcus*	Rapid colonization by breast milk-adapted bacteria, especially *Bifidobacterium*	BF, antibiotic exposure, home environment
6 Months	*Bifidobacterium*, *Clostri-dium*, *Veillonella*	Increased diversity; introduction of solid foods begins shaping microbial complexity	Introduction of complementary foods, feeding type
1 Year	*Bacteroides*, *Clostridium*, *Prevotella*	*Firmicutes* and *Bacteroidetes* start to dominate; gut becomes more anaerobic	Dietary diversity, cessation of BF
3 Years	*Bacteroides*, *Clostridium*, *Prevotella*, *Faecalibacterium*, *Ruminococcus*, *Akkermansia*	Microbiota reaches adult-like composition; stable and diverse community	Long-term diet, environment, early-life exposures

**Table 3:** Sources of variability across infant microbiome studies.

Factors	Potential Impact	References
Sequencing method	Different taxonomic resolution	[116]
Sampling age	Microbiota rapidly changes in infancy	[114]
Definition of BF	Exclusive vs. partial	[51,117]
Formula composition	Protein, HMOs, probiotic content	[51]
Geographic variation	Diet, environment etc.	[40]
Bioinformatics pipelines	Taxonomic classification differences	[40]

## Data Availability

No new data were created or analyzed in this study.

## References

[1] Ziegler E.E. (2006). Growth of Breast-Fed and Formula-Fed Infants. Proceedings of the 58th Nestlé Nutrition Workshop, Pediatric Program, Ho Chi Minh, Vietnam.

[2] Eidelman A.I., Schanler R.J., Johnston M., Landers S., Noble L., Szucs K., Viehmann L. (2012). Breastfeeding and the Use of Human Milk. Pediatrics.

[3] Lessen R., Kavanagh K. (2015). Position of the Academy of Nutrition and Dietetics: Promoting and Supporting Breastfeeding. J. Acad. Nutr. Diet..

[4] Martin R.M., Kramer M.S., Patel R., Rifas-Shiman S.L., Thompson J., Yang S., Vilchuck K., Bogdanovich N., Hameza M., Tilling K. (2017). Effects of Promoting Long-Term, Exclusive Breastfeeding on Adolescent Adiposity, Blood Pressure, and Growth Trajectories: A Secondary Analysis of a Randomized Clinical Trial. JAMA Pediatr..

[5] Owen C.G., Martin R.M., Whincup P.H., Smith G.D., Cook D.G. (2006). Does Breastfeeding Influence Risk of Type 2 Diabetes in Later Life? A Quantitative Analysis of Published Evidence. Am. J. Clin. Nutr..

[6] Oddy W.H. (2013). Longer Duration of Exclusive Breastfeeding Associated with Reduced Risk of Childhood Asthma up to Age Six. Evid. Based Nurs..

[7] Trofin F., Cianga P., Constantinescu D., Iancu L.S., Iancu R.I., Păduraru D., Nastase E.V., Buzilă E.R., Luncă C., Cianga C.M. (2025). The Legacy of COVID-19 in Breast Milk: The Association of Elevated Anti-Inflammatory and Antimicrobial Proteins with Vaccination or Infection. Curr. Issues Mol. Biol..

[8] Trofin F., Nastase E.V., Iancu L.S., Constantinescu D., Cianga C.M., Luncă C., Ursu R.G., Cianga P., Dorneanu O.S. (2022). Anti-RBD IgA and IgG Response and Transmission in Breast Milk of Anti-SARS-CoV-2 Vaccinated Mothers. Pathogens.

[9] Trofin F., Dorneanu O.S., Constantinescu D., Nastase E.V., Luncă C., Iancu L.S., Andrioaie I.-M., Duhaniuc A., Cianga C.M., Pavel-Tanasa M. (2022). Cytokines and Chemokines in Breastmilk of SARS-CoV-2 Infected or COVID-19 Vaccinated Mothers. Vaccines.

[10] Kramer M.S., Aboud F., Mironova E., Vanilovich I., Platt R.W., Matush L., Igumnov S., Fombonne E., Bogdanovich N., Ducruet T. (2008). Breastfeeding and Child Cognitive Development: New Evidence from a Large Randomized Trial. Arch. Gen. Psychiatry.

[11] Nyaradi A., Oddy W.H., Hickling S., Li J., Foster J.K. (2015). The Relationship between Nutrition in Infancy and Cognitive Performance during Adolescence. Front. Nutr..

[12] World Health Organization Infant and Young Child Nutrition. http://apps.who.int/gb/archive/pdf_files/WHA55/ea5515.pdf.

[13] Savino F., Bebetti S., Lignori S.A., Sorrenti M., Cordero Di Montezemolo L. (2013). Advances on Human Milk Hormones and Protection against Obesity. Cell. Mol. Biol..

[14] Stevens E.E., Patrick T.E., Pickler R. (2009). A History of Infant Feeding. J. Perinat. Educ..

[15] Oddy W.H. (2002). The Impact of Breastmilk on Infant and Child Health. Breastfeed. Rev..

[16] Kramer M.S., Guo T., Platt R.W., Shapiro S., Collet J.P., Chalmers B., Hodnett E., Sevkovskaya Z., Dzikovich I., Vanilovich I. (2002). Breastfeeding and Infant Growth: Biology or Bias?. Pediatrics.

[17] Bomer-Norton C. (2014). Breastfeeding: A Holistic Concept Analysis. Public Health Nurs..

[18] Sacker A., Quigley M.A., Kelly Y.J. (2006). Breastfeeding and Developmental Delay: Findings from the Millennium Cohort Study. Pediatrics.

[19] Der G., Batty G.D., Deary I.J. (2006). Effect of Breast Feeding on Intelligence in Children: Prospective Study, Sibling Pairs Analysis, and Meta-Analysis. BMJ.

[20] Zhou S.J., Baghurst P., Gibson R.A., Makrides M. (2007). Home Environment, Not Duration of Breastfeeding, Predicts Intelligence Quotient of Children at Four Years. Nutrition.

[21] Gale C., Logan K.M., Santhakumaran S., Parkinson J.R.C., Hyde M.J., Modi N. (2012). Effect of Breastfeeding Compared with Formula Feeding on Infant Body Composition: A Systematic Review and Meta-Analysis. Am. J. Clin. Nutr..

[22] Thursby E., Juge N. (2017). Introduction to the Human Gut Microbiota. Biochem. J..

[23] Al Bander Z., Nitert M.D., Mousa A., Naderpoor N. (2020). The Gut Microbiota and Inflammation: An Overview. Int. J. Environ. Res. Public Health.

[24] de Vos W.M., Tilg H., Van Hul M., Cani P.D. (2022). Gut Microbiome and Health: Mechanistic Insights. Gut.

[25] Brushett S., Sinha T., Reijneveld S.A., de Kroon M.L.A., Zhernakova A. (2020). The Effects of Urbanization on the Infant Gut Microbiota and Health Outcomes. Front. Pediatr..

[26] Nielsen C.C., Gascon M., Osornio-Vargas A.R., Shier C., Guttman D.S., Becker A.B., Azad M.B., Sears M.R., Lefebvre D.L., Moraes T.J. (2020). Natural environments in the urban context and gut microbiota in infants. Environ. Int..

[27] Finderle J., Schleicher V.S., Schleicher L.M.S., Krsek A., Braut T., Baticic L. (2026). Exercise-Induced Modulation of the Gut Microbiota: Mechanisms, Evidence, and Implications for Athlete Health. Gastrointest. Disord..

[28] Varghese S., Rao S., Khattak A., Zamir F., Chaari A. (2024). Physical Exercise and the Gut Microbiome: A Bidirectional Relationship Influencing Health and Performance. Nutrients.

[29] Mailing L.J., Allen J.M., Buford T.W., Fields C.J., Woods J.A. (2019). Exercise and the Gut Microbiome: A Review of the Evidence, Potential Mechanisms, and Implications for Human Health. Exerc. Sport Sci. Rev..

[30] Beurel E. (2024). Stress in the microbiome-immune crosstalk. Gut Microbes.

[31] Sejbuk M., Siebieszuk A., Witkowska A.M. (2024). The Role of Gut Microbiome in Sleep Quality and Health: Dietary Strategies for Microbiota Support. Nutrients.

[32] Sun J., Fang D., Wang Z., Liu Y. (2023). Sleep Deprivation and Gut Microbiota Dysbiosis: Current Understandings and Implications. Int. J. Mol. Sci..

[33] Lin Z., Jiang T., Chen M., Ji X., Wang Y. (2024). Gut microbiota and sleep: Interaction mechanisms and therapeutic prospects. Open Life Sci..

[34] Rinninella E., Raoul P., Cintoni M., Franceschi F., Miggiano G.A.D., Gasbarrini A., Mele M.C. (2019). What Is the Healthy Gut Microbiota Composition? A Changing Ecosystem across Age, Environment, Diet, and Diseases. Microorganisms.

[35] Xie R., Sun Y., Wu J., Huang S., Jin G., Guo Z., Zhang Y., Liu T., Liu X., Cao X. (2018). Maternal High-Fat Diet Alters Gut Microbiota of Offspring and Exacerbates DSS-Induced Colitis in Adulthood. Front. Immunol..

[36] Bokulich N.A., Chung J., Battaglia T., Henderson N., Jay M., Li H., Lieber A.D., Wu F., Perez-Perez G.I., Chen Y. (2016). Antibiotics, Birth Mode, and Diet Shape Microbiome Maturation during Early Life. Sci. Transl. Med..

[37] Baker P., Santos T., Neves P.A.R., Machado P., Smith J., Piwoz E., Barros A.J.D., Victora C.G., McCoy D. (2021). First-Food Systems Transformations and the Ultra-Processing of Infant and Young Child Diets: The Determinants, Dynamics and Consequences of the Global Rise in Commercial Milk Formula Consumption. Matern. Child Nutr..

[38] Catassi G., Aloi M., Giorgio V., Gasbarrini A., Cammarota G., Ianiro G. (2024). The Role of Diet and Nutritional Interventions for the Infant Gut Microbiome. Nutrients.

[39] Le Doare K., Holder B., Bassett A., Pannaraj P.S. (2018). Mother’s Milk: A Purposeful Contribution to the Development of the Infant Microbiota and Immunity. Front. Immunol..

[40] Yatsunenko T., Rey F.E., Manary M.J., Trehan I., Dominguez-Bello M.G., Contreras M., Magris M., Hidalgo G., Baldassano R.N., Anokhin A.P. (2012). Human gut microbiome viewed across age and geography. Nature.

[41] Milani C., Duranti S., Bottacini F., Casey E., Turroni F., Mahony J., Belzer C., Delgado Palacio S., Arboleya Montes S., Mancabelli L. (2017). The First Microbial Colonizers of the Human Gut: Composition, Activities, and Health Implications of the Infant Gut Microbiota. Microbiol. Mol. Biol. Rev..

[42] Matsuki T., Yahagi K., Mori H., Matsumoto H., Hara T., Tajima S., Ogawa E., Kodama H., Yamamoto K., Yamada T. (2016). A Key Genetic Factor for Fucosyllactose Utilization Affects Infant Gut Microbiota Development. Nat. Commun..

[43] Goodrich J.K., Waters J.L., Poole A.C., Sutter J.L., Koren O., Blekhman R., Beaumont M., Van Treuren W., Knight R., Bell J.T. (2014). Human genetics shape the gut microbiome. Cell.

[44] Pantazi A.C., Balasa A.L., Mihai C.M., Chisnoiu T., Lupu V.V., Kassim M.A.K., Mihai L., Frecus C.E., Chirila S.I., Lupu A. (2023). Development of Gut Microbiota in the First 1000 Days after Birth and Potential Interventions. Nutrients.

[45] Vallès Y., Artacho A., Pascual-García A., Ferrús M.L., Gosalbes M.J., Abellán J.J., Francino M.P. (2014). Microbial Succession in the Gut: Directional Trends of Taxonomic and Functional Change in a Birth Cohort of Spanish Infants. PLoS Genet..

[46] Ardissone A.N., de la Cruz D.M., Davis-Richardson A.G., Rechcigl K.T., Li N., Drew J.C., Murgas-Torrazza R., Sharma R., Hudak M.L., Triplett E.W. (2014). Meconium Microbiome Analysis Identifies Bacteria Correlated with Premature Birth. PLoS ONE.

[47] Dominguez-Bello M.G., Costello E.K., Contreras M., Magris M., Hidalgo G., Fierer N., Knight R. (2010). Delivery mode shapes the acquisition and structure of the initial microbiota across multiple body habitats in newborns. Proc. Natl. Acad. Sci. USA.

[48] Hermansson H., Kumar H., Collado M.C., Salminen S., Isolauri E., Rautava S. (2019). Breast Milk Microbiota Is Shaped by Mode of Delivery and Intrapartum Antibiotic Exposure. Front. Nutr..

[49] Lemas D.J., Yee S., Cacho N., Miller D., Cardel M., Gurka M., Janicke D., Shenkman E. (2016). Exploring the contribution of maternal antibiotics and breastfeeding to development of the infant microbiome and pediatric obesity. Semin. Fetal Neonatal Med..

[50] Yasmin F., Tun H.M., Konya T., Guttman D.S., Chari R.S., Field C.J., Becker A.B., Mandhane P.J., Turvey S.E., Subbarao P. (2017). Cesarean Section, Formula Feeding, and Infant Antibiotic Exposure: Separate and Combined Impacts on Gut Microbial Changes in Later Infancy. Front. Pediatr..

[51] Berger B., Porta N., Foata F., Grathwohl D., Delley M., Moine D., Charpagne A., Siegwald L., Descombes P., Alliet P. (2020). Linking Human Milk Oligosaccharides, Infant Fecal Community Types, and Later Risk To Require Antibiotics. mBio.

[52] Jones C. (2001). Maternal Transmission of Infectious Pathogens in Breast Milk. J. Paediatr. Child Health.

[53] Kebbe M., Leung K., Perrett B., Reimer R.A., Adamo K., Redman L.M. (2025). Effects of Infant Formula Supplemented with Prebiotics on the Gut Microbiome, Gut Environment, Growth Parameters, and Safety and Tolerance: A Systematic Review and Meta-Analysis. Nutr. Rev..

[54] Borewicz K., Suarez-Diez M., Hechler C., Beijers R., de Weerth C., Arts I., Penders J., Thijs C., Nauta A., Lindner C. (2019). The effect of prebiotic fortified infant formulas on microbiota composition and dynamics in early life. Sci. Rep..

[55] Hugon P., Dufour J.C., Colson P., Fournier P.E., Sallah K., Raoult D. (2015). A Comprehensive Repertoire of Prokaryotic Species Identified in Human Beings. Lancet Infect. Dis..

[56] Li J., Jia H., Cai X., Zhong H., Feng Q., Sunagawa S., Arumugam M., Kultima J.R., Prifti E., Nielsen T. (2014). An Integrated Catalog of Reference Genes in the Human Gut Microbiome. Nat. Biotechnol..

[57] Donaldson G.P., Lee S.M., Mazmanian S.K. (2015). Gut Biogeography of the Bacterial Microbiota. Nat. Rev. Microbiol..

[58] Macpherson A.J., McCoy K.D. (2013). Stratification and Compartmentalisation of Immunoglobulin Responses to Commensal Intestinal Microbes. Semin. Immunol..

[59] Gu S., Chen D., Zhang J.-N., Lv X., Wang K., Duan L.P., Nie Y., Wu X.L. (2013). Bacterial Community Mapping of the Mouse Gastrointestinal Tract. PLoS ONE.

[60] Hansen C.H.F., Nielsen D.S., Kverka M., Zakostelska Z., Klimesova K., Hudcovic T., Tlaskalova-Hogenova H., Hansen A.K. (2012). Patterns of Early Gut Colonization Shape Future Immune Responses of the Host. PLoS ONE.

[61] Chung H., Pamp S.J., Hill J.A., Surana N.K., Edelman S.M., Troy E.B., Reading N.C., Villablanca E.J., Wang S., Mora J.R. (2012). Gut Immune Maturation Depends on Colonization with a Host-Specific Microbiota. Cell.

[62] Koren O., Goodrich J.K., Cullender T.C., Spor A., Laitinen K., Bäckhed H.K., Gonzalez A., Werner J.J., Angenent L.T., Knight R. (2012). Host Remodeling of the Gut Microbiome and Metabolic Changes during Pregnancy. Cell.

[63] Makino H., Kushiro A., Ishikawa E., Muylaert D., Kubota H., Sakai T., Oishi K., Martin R., Ben Amor K., Oozeer R. (2011). Transmission of Intestinal *Bifidobacterium longum* subsp. *longum* Strains from Mother to Infant, Determined by Multilocus Sequencing Typing and Amplified Fragment Length Polymorphism. Appl. Environ. Microbiol..

[64] Martín R., Langa S., Reviriego C., Jimínez E., Marín M.L., Xaus J., Fernández L., Rodríguez J.M. (2003). Human Milk Is a Source of Lactic Acid Bacteria for the Infant Gut. J. Pediatr..

[65] Matsumiya Y., Kato N., Watanabe K., Kato H. (2002). Molecular Epidemiological Study of Vertical Transmission of Vaginal *Lactobacillus* Species from Mothers to Newborn Infants in Japanese, by Arbitrarily Primed Polymerase Chain Reaction. J. Infect. Chemother..

[66] Clemente J.C., Ursell L.K., Parfrey L.W., Knight R. (2012). The Impact of the Gut Microbiota on Human Health: An Integrative View. Cell.

[67] Mitsuoka T. (2014). Development of Functional Foods. Biosci. Microbiota Food Health.

[68] Yao Y., Cai X., Ye Y., Wang F., Chen F., Zheng C. (2021). The Role of Microbiota in Infant Health: From Early Life to Adulthood. Front. Immunol..

[69] Martin C.R., Ling P.R., Blackburn G.L. (2016). Review of Infant Feeding: Key Features of Breast Milk and Infant Formula. Nutrients.

[70] Ballard O., Morrow A.L. (2013). Human Milk Composition: Nutrients and Bioactive Factors. Pediatr. Clin. N. Am..

[71] Mosca F., Giannì M.L. (2017). Human Milk: Composition and Health Benefits. Pediatr. Med. Chir..

[72] Agostoni C., Braegger C., Decsi T., Kolacek S., Koletzko B., Michaelsen K.F., Mihatsch W., Moreno L.A., Puntis J., ESPGHAN Committee on Nutrition (2009). Breast-Feeding: A Commentary by the ESPGHAN Committee on Nutrition. J. Pediatr. Gastroenterol. Nutr..

[73] Eriksen K.G., Christensen S.H., Lind M.V., Michaelsen K.F. (2018). Human Milk Composition and Infant Growth. Curr. Opin. Clin. Nutr. Metab. Care.

[74] De Luca A., Hankard R., Alexandre-Gouabau M.C., Ferchaud-Roucher V., Darmaun D., Boquien C.Y. (2016). Higher Concentrations of Branched-Chain Amino Acids in Breast Milk of Obese Mothers. Nutrition.

[75] Lönnerdal B. (2004). Human Milk Proteins: Key Components for the Biological Activity of Human Milk. Adv. Exp. Med. Biol..

[76] Thurl S., Munzert M., Boehm G., Matthews C., Stahl B. (2017). Systematic Review of the Concentrations of Oligosaccharides in Human Milk. Nutr. Rev..

[77] Moukarzel S., Bode L. (2017). Human Milk Oligosaccharides and the Preterm Infant: A Journey in Sickness and in Health. Clin. Perinatol..

[78] Plaza-Díaz J., Fontana L., Gil A. (2018). Human Milk Oligosaccharides and Immune System Development. Nutrients.

[79] Gavin A., Ostovar K. (1977). Microbiological Characterization of Human Milk. J. Food Prot..

[80] Martín R., Jiménez E., Heilig H., Fernández L., Marín M.L., Zoetendal E.G., Rodríguez J.M. (2009). Isolation of Bifidobacteria from Breast Milk and Assessment of the Bifidobacterial Population by PCR-Denaturing Gradient Gel Electrophoresis and Quantitative Real-Time PCR. Appl. Environ. Microbiol..

[81] Jost T., Lacroix C., Braegger C., Chassard C. (2013). Assessment of Bacterial Diversity in Breast Milk Using Culture-Dependent and Culture-Independent Approaches. Br. J. Nutr..

[82] Hunt K.M., Foster J.A., Forney L.J., Schütte U.M., Beck D.L., Abdo Z., Fox L.K., Williams J.E., McGuire M.K., McGuire M.A. (2011). Characterization of the Diversity and Temporal Stability of Bacterial Communities in Human Milk. PLoS ONE.

[83] Murphy K., Curley D., O’Callaghan T.F., O’Shea C.A., Dempsey E.M., O’Toole P.W., Ross R.P., Ryan C.A., Stanton C. (2017). The Composition of Human Milk and Infant Faecal Microbiota over the First Three Months of Life: A Pilot Study. Sci. Rep..

[84] Moossavi S., Sepehri S., Robertson B., Bode L., Goruk S., Field C.J., Lix L.M., de Souza R.J., Becker A.B., Mandhane P.J. (2019). Composition and Variation of the Human Milk Microbiota Are Influenced by Maternal and Early-Life Factors. Cell Host Microbe.

[85] Shin D.Y., Park J., Yi D.Y. (2021). Comprehensive Analysis of the Effect of Probiotic Intake by the Mother on Human Breast Milk and Infant Fecal Microbiota. J. Korean Med. Sci..

[86] Milani C., Mancabelli L., Lugli G.A., Duranti S., Turroni F., Ferrario C., Mangifesta M., Viappiani A., Ferretti P., Gorfer V. (2015). Exploring Vertical Transmission of *Bifidobacteria* from Mother to Child. Appl. Environ. Microbiol..

[87] Yi D.Y., Kim S.Y. (2021). Human Breast Milk Composition and Function in Human Health: From Nutritional Components to Microbiome and MicroRNAs. Nutrients.

[88] Bäckhed F., Roswall J., Peng Y., Feng Q., Jia H., Kovatcheva-Datchary P., Li Y., Xia Y., Xie H., Zhong H. (2015). Dynamics and Stabilization of the Human Gut Microbiome during the First Year of Life. Cell Host Microbe.

[89] Pannaraj P.S., Li F., Cerini C., Bender J.M., Yang S., Rollie A., Adisetiyo H., Zabih S., Lincez P.J., Bittinger K. (2017). Association between Breast Milk Bacterial Communities and Establishment and Development of the Infant Gut Microbiome. JAMA Pediatr..

[90] Wall R., Ross R.P., Ryan C.A., Hussey S., Murphy B., Fitzgerald G.F., Stanton C. (2009). Role of Gut Microbiota in Early Infant Development. Clin. Med. Pediatr..

[91] Sandall J., Tribe R.M., Avery L., Mola G., Visser G.H., Homer C.S., Gibbons D., Kelly N.M., Kennedy H.P., Kidanto H. (2018). Short-Term and Long-Term Effects of Caesarean Section on the Health of Women and Children. Lancet.

[92] Tirone C., Pezza L., Paladini A., Tana M., Aurilia C., Lio A., D’Ippolito S., Tersigni C., Posteraro B., Sanguinetti M. (2019). Gut and Lung Microbiota in Preterm Infants: Immunological Modulation and Implication in Neonatal Outcomes. Front. Immunol..

[93] Fehr K., Moossavi S., Sbihi H., Boutin R.C.T., Bode L., Robertson B., Yonemitsu C., Field C.J., Becker A.B., Mandhane P.J. (2020). Breastmilk Feeding Practices Are Associated with the Co-Occurrence of Bacteria in Mothers’ Milk and the Infant Gut: The CHILD Cohort Study. Cell Host Microbe.

[94] Bakshi S., Paswan V.K., Yadav S.P., Bhinchhar B.K., Kharkwal S., Rose H., Kanetkar P., Kumar V., Al-Zamani Z.A.S., Bunkar D.S. (2023). A comprehensive review on infant formula: Nutritional and functional constituents, recent trends in processing and its impact on infants’ gut microbiota. Front. Nutr..

[95] Laursen M.F., Bahl M.I., Michaelsen K.F., Licht T.R. (2017). First Foods and Gut Microbes. Front. Microbiol..

[96] Ho N.T., Li F., Lee-Sarwar K.A., Tun H.M., Brown B.P., Pannaraj P.S., Bender J.M., Azad M.B., Thompson A.L., Weiss S.T. (2018). Meta-Analysis of Effects of Exclusive Breastfeeding on Infant Gut Microbiota across Populations. Nat. Commun..

[97] Thompson A.L., Monteagudo-Mera A., Cadenas M.B., Lampl M.L., Azcarate-Peril M.A. (2015). Milk- and Solid-Feeding Practices and Daycare Attendance Are Associated with Differences in Bacterial Diversity, Predominant Communities, and Metabolic and Immune Function of the Infant Gut Microbiome. Front. Cell. Infect. Microbiol..

[98] Madan J.C., Hoen A.G., Lundgren S.N., Farzan S.F., Cottingham K.L., Morrison H.G., Sogin M.L., Li H., Moore J.H., Karagas M.R. (2016). Association of Cesarean Delivery and Formula Supplementation with the Intestinal Microbiome of 6-Week-Old Infants. JAMA Pediatr..

[99] Vael C., Verhulst S.L., Nelen V., Goossens H., Desager K.N. (2011). Intestinal Microflora and Body Mass Index during the First Three Years of Life: An Observational Study. Gut Pathog..

[100] Masum A.K.M., Chandrapala J., Huppertz T., Adhikari B., Zisu B. (2021). Production and Characterization of Infant Milk Formula Powders: A Review. Dry. Technol..

[101] Herrera-Quintana L., Vázquez-Lorente H., Hinojosa-Nogueira D., Plaza-Díaz J. (2024). Relationship between Infant Feeding and the Microbiome: Implications for Allergies and Food Intolerances. Children.

[102] Dipasquale V., Serra G., Corsello G., Romano C. (2020). Standard and Specialized Infant Formulas in Europe: Making, Marketing, and Health Outcomes. Nutr. Clin. Pract..

[103] Fenelon M.A., Hickey R.M., Buggy A., McCarthy N., Murphy E.G., Deeth H.C., Bansal N. (2019). Whey Proteins in Infant Formula. Whey Proteins: From Milk to Medicine.

[104] Sjöblad S. (2019). Could the High Consumption of High Glycaemic Index Carbohydrates and Sugars, Associated with the Nutritional Transition to the Western Type of Diet, Be the Common Cause of the Obesity Epidemic and the Worldwide Increasing Incidences of Type 1 and Type 2 Diabetes?. Med. Hypotheses.

[105] Mazzocchi A., D’Oria V., De Cosmi V., Bettocchi S., Milani G.P., Silano M., Agostoni C. (2018). The Role of Lipids in Human Milk and Infant Formulae. Nutrients.

[106] Inchingolo F., Inchingolo A.M., Latini G., Ferrante L., de Ruvo E., Campanelli M., Longo M., Palermo A., Inchingolo A.D., Dipalma G. (2024). Difference in the Intestinal Microbiota between Breastfed Infants and Infants Fed with Artificial Milk: A Systematic Review. Pathogens.

[107] Guaraldi F., Salvatori G. (2012). Effect of Breast and Formula Feeding on Gut Microbiota Shaping in Newborns. Front. Cell. Infect. Microbiol..

[108] Davis E.C., Castagna V.P., Sela D.A., Hillard M.A., Lindberg S., Mantis N.J., Seppo A.E., Järvinen K.M. (2022). Gut microbiome and breast-feeding: Implications for early immune development. J. Allergy Clin. Immunol..

[109] Timby N., Domellöf E., Hernell O., Lönnerdal B., Domellöf M. (2014). Neurodevelopment, nutrition, and growth until 12 mo of age in infants fed a low-energy, low-protein formula supplemented with bovine milk fat globule membranes: A randomized controlled trial. Am. J. Clin. Nutr..

[110] Bode L. (2015). The functional biology of human milk oligosaccharides. Early Hum. Dev..

[111] Koletzko B., Baker S., Cleghorn G., Neto U.F., Gopalan S., Hernell O., Hock Q.S., Jirapinyo P., Lonnerdal B., Pencharz P. (2005). Global standard for the composition of infant formula: Recommendations of an ESPGHAN coordinated international expert group. J. Pediatr. Gastroenterol. Nutr..

[112] Puccio G., Alliet P., Cajozzo C., Janssens E., Corsello G., Sprenger N., Wernimont S., Egli D., Gosoniu L., Steenhout P. (2017). Effects of Infant Formula with Human Milk Oligosaccharides on Growth and Morbidity: A Randomized Multicenter Trial. J. Pediatr. Gastroenterol. Nutr..

[113] Gungor D., Nadaud P., LaPergola C.C., Dreibelbis C., Wong Y.P., Terry N., Abrams S.A., Beker L., Jacobovits T., Järvinen K.M. (2019). Infant Milk-Feeding Practices and Food Allergies, Allergic Rhinitis, Atopic Dermatitis, and Asthma throughout the Life Span: A Systematic Review. Am. J. Clin. Nutr..

[114] Stewart C.J., Ajami N.J., O’Brien J.L., Hutchinson D.S., Smith D.P., Wong M.C., Ross M.C., Lloyd R.E., Doddapaneni H., Metcalf G.A. (2018). Temporal development of the gut microbiome in early childhood from the TEDDY study. Nature.

[115] Bode L. (2012). Human milk oligosaccharides: Every baby needs a sugar mama. Glycobiology.

[116] Knight R., Vrbanac A., Taylor B.C., Aksenov A., Callewaert C., Debelius J., Gonzalez A., Kosciolek T., McCall L.I., McDonald D. (2018). Best practices for analysing microbiomes. Nat. Rev. Microbiol..

[117] Forbes J.D., Azad M.B., Vehling L., Tun H.M., Konya T.B., Guttman D.S., Field C.J., Lefebvre D., Sears M.R., Becker A.B. (2018). Association of Exposure to Formula in the Hospital and Subsequent Infant Feeding Practices with Gut Microbiota and Risk of Overweight in the First Year of Life. JAMA Pediatr..

[118] Seppo A.E., Bu K., Jumabaeva M., Thakar J., Choudhury R.A., Yonemitsu C., Bode L., Martina C.A., Allen M., Tamburini S. (2021). Infant Gut Microbiome Is Enriched with *Bifidobacterium longum* ssp. infantis in Old Order Mennonites with Traditional Farming Lifestyle. Allergy.

[119] Conta G., Del Chierico F., Reddel S., Marini F., Sciubba F., Capuani G., Tomassini A., Di Cocco M.E., Laforgia N., Baldassarre M.E. (2021). Longitudinal Multi-Omics Study of a Mother-Infant Dyad from Breastfeeding to Weaning: An Individualized Approach to Understand the Interactions Among Diet, Fecal Metabolome and Microbiota Composition. Front. Mol. Biosci..

[120] Du B., Shama A., Zhang Y., Chen B., Bu Y., Chen P.A., Lin C., Liu J., Zheng J., Li Z. (2025). Gut microbiota and plasma metabolites in pregnant mothers and infant atopic dermatitis: A multi-omics study. World Allergy Organ. J..

[121] Barker-Tejeda T.C., Zubeldia-Varela E., Macías-Camero A., Alonso L., Martín-Antoniano I.A., Rey-Stolle M.F., Mera-Berriatua L., Bazire R., Cabrera-Freitag P., Shanmuganathan M. (2024). Comparative characterization of the infant gut microbiome and their maternal lineage by a multi-omics approach. Nat. Commun..

[122] Stolberg-Mathieu G., Mikkelsen L.S., Gottlieb A.D., Mølgaard C., Roager H.M. (2025). The MOTILITY Mother-Child Cohort: A Danish prospective longitudinal cohort study of the infant gut microbiome, nutrition and bowel habits—A study protocol. BMJ Open.

[123] Zhao M., Li X., Li Y., Ma L., Liu Y., Wang S., Tian J., Liang Y., Shen C., Ma X. (2025). Multi-Omics Profiling of the 42-Day Infant Gut as a Pilot Predictor of Atopic Dermatitis at One Year: A Birth Cohort Study in China. J. Inflamm. Res..

[124] Ricci L., Heidrich V., Punčochář M., Armanini F., Ciciani M., Nabinejad A., Fazaeli F., Piperni E., Servais C., Pinto F. (2026). Baby-to-baby strain transmission shapes the developing gut microbiome. Nature.

[125] Ding M., Ross R.P., Dempsey E., Li B., Stanton C. (2025). Infant gut microbiome reprogramming following introduction of solid foods (weaning). Gut Microbes.

[126] Vogel S.C., Murgueitio N., Huth N., Sem K., Knickmeyer R.C., Short S.J., Mills-Koonce R., Propper C., Wagner N.J. (2025). Longitudinal associations between the infant gut microbiome and negative affect in toddlerhood. Dev. Psychopathol..

